# A Supervised Machine Learning Approach to Detect the On/Off State in Parkinson’s Disease Using Wearable Based Gait Signals

**DOI:** 10.3390/diagnostics10060421

**Published:** 2020-06-20

**Authors:** Satyabrata Aich, Jinyoung Youn, Sabyasachi Chakraborty, Pyari Mohan Pradhan, Jin-han Park, Seongho Park, Jinse Park

**Affiliations:** 1 Terenz Co., Limited, Busan 48060, Korea; james@sikonic.io (S.A.); c.sabyasachi99@gmail.com (S.C.); 2 Department of Neurology, Samsung Medical Center, School of medicine Sungkyunkwan University, Seoul 06351, Korea; 3 Department of Electronics and Communication Engineering, IIT Roorkee 247667, India; pmpradhan.fec@iitr.ac.in; 4 Department of Respiratory Medicine, Haeundae Paik Hospital, Inje University, Busan 48108, Korea; h00389@paik.ac.kr; 5 Department of Neurology, Haeundae Paik Hospital, Inje University, Busan 48108 Korea; risepsh@gmail.com

**Keywords:** Parkinson’s disease, medication state, machine learning, wearable device, “On” and “Off”

## Abstract

Fluctuations in motor symptoms are mostly observed in Parkinson’s disease (PD) patients. This characteristic is inevitable, and can affect the quality of life of the patients. However, it is difficult to collect precise data on the fluctuation characteristics using self-reported data from PD patients. Therefore, it is necessary to develop a suitable technology that can detect the medication state, also termed the “On”/“Off” state, automatically using wearable devices; at the same time, this could be used in the home environment. Recently, wearable devices, in combination with powerful machine learning techniques, have shown the potential to be effectively used in critical healthcare applications. In this study, an algorithm is proposed that can detect the medication state automatically using wearable gait signals. A combination of features that include statistical features and spatiotemporal gait features are used as inputs to four different classifiers such as random forest, support vector machine, K nearest neighbour, and Naïve Bayes. In total, 20 PD subjects with definite motor fluctuations have been evaluated by comparing the performance of the proposed algorithm in association with the four aforementioned classifiers. It was found that random forest outperformed the other classifiers with an accuracy of 96.72%, a recall of 97.35%, and a precision of 96.92%.

## 1. Introduction

Parkinson’s disease (PD) is the second most common neurodegenerative disease that presents with motor symptoms such as bradykinesia, tremor, and rigidity. The prognosis of PD is composed of several stages including the honeymoon period, the motor complication stage, and drug-resistant stage. In the honeymoon period, the severity of motor symptoms is uniform/constant throughout the day. However, in the motor complication stage, motor symptoms fluctuate as the effect of medication remains for a small time duration. Therefore, most clinical and research studies focus on recognizing and stabilizing fluctuating motor symptoms in the motor complication stage. 

Dopaminergic medication improves motor symptoms in the early stages. However, motor fluctuations appear when the disease progresses [1,2,3]. Motor fluctuation is defined based on the reduction in “On” time and increase in “Off” time in PD patients. The “On” state is defined as the time duration over which the effect of dopaminergic medicine is present in PD patients. In the same context, the “Off” state is defined as the time duration over which the effect of dopaminergic medicine is absent in the patients. The reduced duration of the “On” time generates more Parkinsonism, which has a significant negative impact on the quality of life in PD patients [4,5,6]. Therefore, even though it is essential to evaluate motor fluctuation for the personalized management of PD patients, it is difficult to estimate the fluctuation in the real-world environment due to the short interview time and fluctuating characteristics of the motor symptoms in PD [7,8,9]. Usually, a self-report diary is used to mesaure the fluctuation phenomenon [10]. However, it is difficult to fill up a diary every 1 or 2 h, especially during the “Off” status because of severe Parkinsonism. Additionally, patients with advanced PD could have severe cognitive declination. Therefore, this method is not able to provide accurate times for “On” and “Off” status in advanced PD patients.

Clinically, most patients do not complain about their symptoms in terms of severity. On the contrary, they categorize the symptoms as “On”/“Off”. It is very difficult for patients to express their symptoms as percentage of severity. Thus, they usually report whether they are currently in “On” status or “Off” status. Additionally, unlike appendicular symptoms, including rigidity or bradykinesia, it is not possible to grade the gait symptoms. Therefore, it is very difficult for PD patients to describe their gait symptoms in terms of severity. 

A lot of studies in the literature focus on detecting the severity of motor symptoms in PD patients. Comparing the severity of motor symptoms across multiple PD patients and across “On”/“Off” states of a single PD patient, it could be observed that there is a high variability in gait features in the latter case which makes the detection, nonlinear classification and prediction of “On”/“Off” states highly challenging. Thus, the current study aims to detect the “On” and “Off” states in a PD patient using gait features.

With the development of wearable technology, an objective assessment of various Parkinsonian symptoms such as gait disorders, tremors, and bradykinesia was made practically feasible in recent years [11]. Previous studies using the wearable device proved to be effective in detecting gait disorders, tremors, and dyskinesia [12,13]. In this paper, the objective of this research is to develop an assessment system using wearable accelerometer-based sensors which can detect the “On” state and “Off” state of PD patients automatically from the raw accelerometer gait signals collected thorugh the wearable accelerometer-based sensors. This kind of assessment system will play a major role in improving the monitoring process of PD in natural environments. It will also provide information about the time duration of the dopaminergic medication effect on PD patients in natural environments that helps to decrease the cost associated with the tests that are done frequently in clinics.

In recent years, machine learning techniques have been applied to wearable sensor data for detecting different symptoms in PD such as gait disorders, tremors, and mediation states. These techniques are also used for the classification of PD groups from healthy control groups as well as from other neurological diseases based on the extracted features from the wearable sensor data [14,15,16,17,18]. In this research work, we propose an algorithm that can automatically detect the “On” and “Off” state of PD using the wearable accelerometer data. To achieve our objective, we have collected accelerometer data during “On” and “Off” status of PD patients who were recruited at Haeundae Paik Hospital and Samsung Medical Center, Korea. Features extracted from those data have been used to train the machine learning classifiers. Furthermore, these classifiers are used to automatically detect the two different states on the new set of test data. Later, these states were validated based on the report provided by the neurologist. The proposed system will be helpful in monitoring the effect of dopaminergic medicine on PD patients in the home environment.

Previous reports have tried to monitor motor fluctuation in PD patients and provided the possibility of the objective measurement of motor complications in PD. However, studies and methods which present technical validation in controlled environments are difficult to incorporate directly in the clinical field. Keijser et al. [19] tried to investigate the accuracy of ambulatory motor assessment by wearable sensors. They demonstrated that wearable sensors can detect motor dyskinesia with high accuracy using frequency based approach. This report aims to achieve the objective monitoring of motor complications in a real-world environment. However, the participants in this report were asked to perform a specific task which does not imitate a perfect real-world environment. We postulate that the gold standard for a free-living environment is difficult to design, and a frequency-based approach has many pitfalls in terms of accuracy due to various kinds of noise. Therefore, we assume that the machine learning method for data analysis might provide an alternative solution for increasing accuracy in real world environment. In the past decade, the machine learning method has been used to detect other symptoms such as tremors and freezing of gait (FoG) in PD patients with acceptable accuracy. This study is performed in a well-controlled laboratory environment with a specific task. We use the machine learning method for the development of an algorithm for gait analysis during “On”/“Off” states in PD patients. We assume that gait performance is the most crucial attribute associated with quality of life, and therefore, we focus on gait disorders, rather than other Parkinsonian symptoms in PD for monitoring “On”/“Off” states in order to satisfy a clinical need.

The organization of the paper is as follows: Section 2 discusses the previous work related to this research. Section 3 focuses on data collection and methods used for analyzing the data in this research. Section 4 shows the results of the proposed method. Section 5 presents the discussion. Section 6 presents the conclusion of this research work.

## 2. Related Work

In the last few years, wearable technology combined with machine learning techniques or statistical techniques has been effective for PD patients because of the nature of the assessment process. These systems have proved to be effective, powerful and also automated so that they can be used in natural environments for improving the quality of life of PD patients. Jeon et al., 2017 [20] proposed an approach to develop an automated system that can classify the severity of tremors using a wearable device consisting of an accelerometer and a gyroscope. This automated system has been developed using five machine learning techniques such as decision tree (DT), k-nearest neighbor (kNN), random forest (RF), and support vector machine (SVM), and discriminant analysis, based on the features extracted from the accelerometer and gyroscope signals. They found that the system is effective with an accuracy of 85.5% and the automated system could be useful for the diagnosis and monitoring of PD patients. The machine learning techniques were used for PD classification, and were explored for the automatic scoring of tremor severity. Samà et al., 2017 [21] proposed a method that can automatically detect gait related disorders in PD patients with bradykinesia using a wearable accelerometer. They collected signals during activities of daily life in the “On” and “Off” states. They used the “Leave One Subject Out (LOSO)” regression model by giving a threshold value to this patient. They used a support vector machine regression model to estimate the severity of bradykinesia with an accuracy of more than 90%. They suggested that the model has the potential to rate bradykinesia in real-life situations. Aich et al., 2018 [22] proposed an approach that can detect FoG in PD patients using wearable devices consisting of an accelerometer. The developed system was an automated system that can classify “FoG” groups from “no FoG” groups based on the extracted features from the accelerometer signals. These features were fed to four different machine learning classifiers such as support vector machine (SVM), Naïve Bayes (NB), k-nearest neighborhood (k-NN), and decision tree (DT) for the classification of “FoG” from “no FoG”. The highest classification accuracy of 88% was obtained with the SVM classifier. It was shown that machine learning techniques combined with wearable sensors could be used to detect falls and for the monitoring of patients in natural environments. Steinmetzer et al., 2019 [23] proposed a method that can detect motor dysfunctions (MD) in PD using a wearable device consisting of accelerometer, gyroscope, and magnetometer. All the subjects were asked to perform the Timed Up and Go test. They used wavelet transformation, Convolutional Neural Network(CNN), and weight voting for the classification of their data. They used a single signal as well as a combination of signals for the classification of data. They found an accuracy of 93.4% when classifying MD from other subjects, and recommended this system for the early detection of MD in real-time environments. Naghavi et al., 2019 [24] proposed a method that can predict “FoG” among PD patients using a wearable device consisting of an accelerometer. The accelerometer signal is labeled using the video captured during the experiment. They used classification algorithms such as SVM, k-NN, and Multilayer Perceptron(MLP) for the prediction of gait events among the patients that have experienced “FoG”. Their model was able to provide an accuracy of 97.4% for the identification of events. They state that the model has the potential to predict gait events accurately with limited event frequency. Baraka et al., 2019 [25] proposed a method that can automatically classify tremors in PD based on the machine learning technique using accelerometer signals and electromyography (EMG).The extracted features from these signals were used as inputs to six different classifiers such as decision tree, k-nearest neighbor, linear discriminant analysis, support vector machine, boosted tree classifiers, and bagged tree classifiers. They found highest accuracy of 99.6% using bagged tree classifiers. Vos et al., 2020 [26] proposed a method that can classify the subjects belonging to PD from Progressive Supranuclear Palsy (PSP) using wearable devices consisting of an accelerometer, gyroscope, and magnetometers. Data were collected using an array of six wearable devices. Features were extracted from the signal data, and used as inputs to the machine learning techniques such as logistic regression (LR) and random forest (RF) applied in this research. It was found that the RF classifier outperformed the LR classifiers while classifying PSP from PD with a sensitivity of 86% and specificity of 90%, and PSP from PD with a sensitivity of 90% and specificity of 97%. They found that the combination of wearable devices and machine learning techniques had the potential to accurately classify PSP and PD, and could be used for detecting different diseases in real-life environments. Ashour et al., 2020 [27] proposed a method that uses data from wearables placed at different locations such as on the hip, knee, and ankles. They used the signals coming from the accelerometer for the detection of “FoG”. They developed this model using a Long Short-Term Memory (LSTM) network, and designed by looking towards patient dependency, then compared this model to a traditional machine learning technique such as a support vector machine (SVM). They found that the LSTM network performed better, with an accuracy of 98.89% compared to SVM, with an accuracy of 80%. Senturk et al., 2020 [28] proposed a model based on feature selection methods and machine learning techniques that can help in the diagnosis of PD at an early stage. They used two different types of feature selection techniques such as recursive feature elimination (RFE) and feature importance. The classification techniques that were used in this study are namely SVM, ANN, and Classifications and Regression Trees. They found that the combination of SVM with RFE performed better, with an accuracy of 93.84%, using a minimum number of voice features. None of the aforementioned studies include the effect of dopaminergic medicine. A summary of the literature surveyed is shown in Table 1.

Keijsers et al. [19] proposed a method that can differentiate between “On” and “Off” states based on daily activities using wearable accelerometers. They used a frequency-based approach for the classification of states by performing 35 functional activities and discussed the possibility of assessing motor complications in real world environments. They found good results in terms of a sensitivity of 0.97 and specificity of 0.97 when distinguishing the two states. Although a frequency-based method produces good results in the unsupervised condition, the effectiveness of this method has largely not been tested in the clinical environment. Patel et al. [29] used a support vector machine to estimate tremor, bradykinesia and dyskinesia based on data collected using accelerometers. The participants in this study were asked to perform specific tasks seven times, and the clinical scores were compared. Although they collected the acceleration data from the upper and lower extremities and were able to monitor the severity of the symptoms, they did not consider gait analysis, which is more accurate and easier for monitoring PD patients. Hoff et al. [30] proposed a method of analyzing the relation between the subjective ratings and objective measures. They used a frequency-based approach for assessing tremors, bradykinesia and dyskinesia by using a wearable accelerometer in different places throughout the body. Since the gold standard method was not considered in this study, subjective rating was used for validation. Although the method was able to distinguish the “On” and “Off” state, the sensitivity and specificity are very low. This method is not feasible in the real-life environment. Rodriguez-Molinero et al. [31] proposed a method that can map gait disturbances using wearable sensors. The participants were asked to perform various tasks in controlled and uncontrolled environments while wearing the sensor around the waist. They found good results in terms of a sensitivity of 0.96 and specificity of 0.94. This study was able to distinguish states with high sensitivity and specificity. Our proposed study is specifically designed for home environments. Furthermore, the proposed method is an automatic process, and can detect the “On” and “Off” states from the raw acceleration signal.

The previous studies outlined above laid a strong foundation for the application of wearable devices in monitoring and diagnosing PD, and the constructive use of classification techniques to automatically detect different states of PD. The proposed method draws much inspiration from the previous literature cited by various researchers. In this study, an algorithm-based method is developed, and validated based on the accelerometer data related to gait by including the signal features as well as spatiotemporal features. This is a unique study that includes features for classifying both the “On” and “Off” states. 

## 3. Materials and Methods

### 3.1. Study Design and Subjects

This study is a cross-sectional, multicenter, observational study. It was carried out at Haeundae Paik Hospital and Samsung Medical Center, South Korea. This study was approved by the institutional review board of each hospital (No. 2019-05-014 and 2019-05-104 for Haeundae Paik Hospital and Samsung Medical Center, respectively), and all the participants have given their consent to participate in this study. The inclusion criteria of PD patients are as follows; (1) patients diagnosed as PD according to Queen’s bank criteria, (2) patients with definite fluctuation, (3) patients who can walk during the “Off” state and, therefore, can perform the task. The exclusion criteria of PD patients are as follows; (1) patients who cannot walk during “Off” state, and therefore, are unable to perform the task, (2) patients who have a disease affecting gait including orthopedic and other neurological diseases, vestibulopathy, and diabetes, (3) patients who have dementia (Korean mini-mental status exam ≤ 24), (4) patients with a psychiatric problem requiring medications, (5) patients with structural brain lesions including white matter changes (age-related white matter change score ≥ 2 on brain MRI), and (5) patients with orthostatic hypotension. For estimating disease severity, we used the Unified Parkinson’s Disease Rating Scale (UPDRS) part III and Hoehn and Yahr staging in “On” and “Off” states. The demographics of PD patients are shown in Table 2. 

### 3.2. Data Collection and Experimental Procedure

In this study, the data were collected from 20 different subjects suffering from PD. Table 2 (above) shows details about the height, weight and age of all the subjects who were enrolled in the data collection procedure. Prior written approval was taken from the above subjects before enrolling them in the data extraction procedure. For the data collection, the patients were asked to perform two courses of “Walking”, one in the “On” state (presence of medication effect) and one in the “Off” state (absence of any medication effect). In the “On” state, patients feel fully comfortable due to the dopaminergic effect. In the “Off” state, patients show Parkinsonian symptoms, and feel uncomfortable, without a dopaminergic effect. We performed the task during “On” state after 1–1.5 h of taking dopaminergic medicine. Similarly, we performed the task during “Off” state after at least 12 h of taking dopaminergic medicine. If the symptoms of patients could not reach the “Off” state after 12 h, patients did not take medicine until they felt uncomfortable. During the data extraction procedure for both the courses, the subjects were fastened with two knee-worn tri-axial accelerometer sensors. The tri-axial accelerometer used in this study was small and lightweight, and had a dimension of 35 mm × 35 mm × 13 mm, and weighed a mere 13.7 g. The sensitivity of the accelerometer sensor used in the data collection procedure was from −8 g to 8 g which was sufficient to measure the movement metric of the subject in all the directions: anterior–posterior, mediolateral, and vertical.

In this study, the data were collected from 20 different subjects suffering from PD. Table 2 (above) shows details about the height, weight and age of all the subjects who were enrolled in the data collection procedure. Prior written approval was taken from the above subjects before enrolling them in the data extraction procedure. For the data collection, the patients were asked to perform two courses of “Walking”, one in the “On” state (presence of medication effect) and one in the “Off” state (absence of any medication effect). In the “On” state, patients feel fully comfortable due to the dopaminergic effect. In the “Off” state, patients show Parkinsonian symptoms, and feel uncomfortable, without a dopaminergic effect. We performed the task during “On” state after 1–1.5 h of taking dopaminergic medicine. Similarly, we performed the task during “Off” state after at least 12 h of taking dopaminergic medicine. If the symptoms of patients could not reach the “Off” state after 12 h, patients did not take medicine until they felt uncomfortable. During the data extraction procedure for both the courses, the subjects were fastened with two knee-worn tri-axial accelerometer sensors. The tri-axial accelerometer used in this study was small and lightweight, and had a dimension of 35 mm × 35 mm × 13 mm, and weighed a mere 13.7 g. The sensitivity of the accelerometer sensor used in the data collection procedure was from −8 g to 8 g which was sufficient to measure the movement metric of the subject in all the directions: anterior–posterior, mediolateral, and vertical.

**Figure 1:**
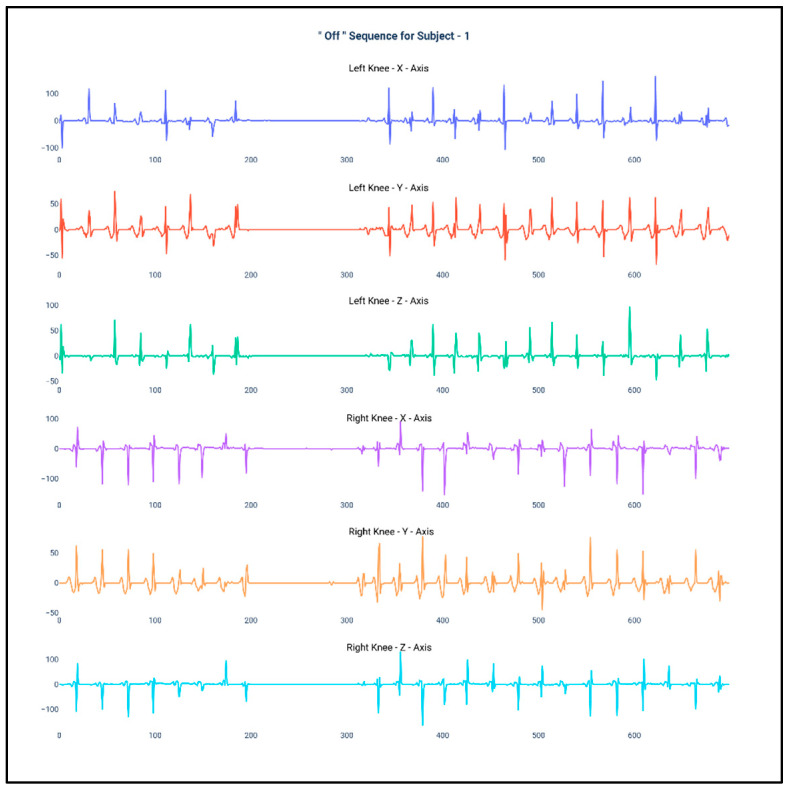
Accelerometer signals of subject 1 for 750 samples during “Off” state.

**Figure 2:**
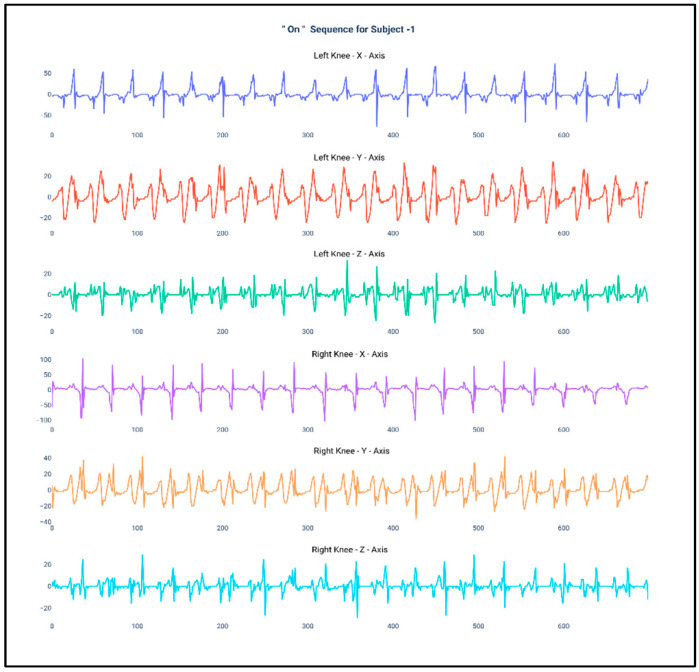
Accelerometer signals of subject 1 for 750 samples during “On” state.

Figure 1 and Figure 2 show the data of Subject 1 for 750 samples of all axes of accelerometer sensors fastened on both the knees and also for both the “Off” and “On” data collection sequence, respectively. As the data is a time series, it is very much required that the stationarity of the dataset needs to be checked. Therefore, the below plots have been generated and, for both the sequences, it can be duly observed that the data maintain proper stationarity with respect to time.

**Figure 3:**
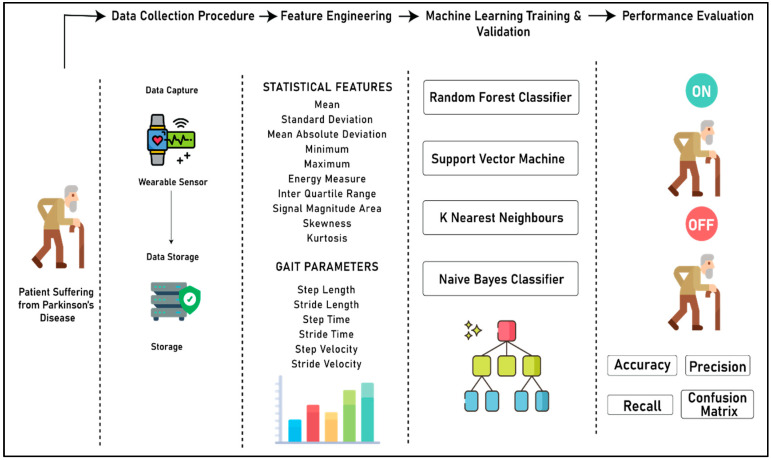
Complete process flow of the experiment.

Figure 3 (below) shows the complete process for the development of the system to detect the “On” and “Off” stages of a patient suffering from PD. The complete flow of the experiment is divided into four different sections, namely the data collection procedure, feature engineering, machine learning model training and validation, and performance evaluation. In the data collection step, the acceleration data from both right and left knee is captured using the wearable sensors, and merged into the database. In the feature engineering step, two different kinds of features are extracted from the accelerometer data, namely statistical features and gait parameters. In the third step, machine learning models are developed by using four different algorithms, namely random forest classifier, support vector machine, k nearest neighbors, and Naïve Bayes classifier. Finally, in the last segment, a qualitative analysis was performed on the predictive power of all four algorithms based on accuracy, precision, recall, and confusion matrix to choose the best classifier for distinguishing the “On” and “Off” states of patients suffering from PD.

The data collection was performed on 20 subjects each, for two courses. During the data collection procedure, the patients were fastened with wearable devices on both knees, which were used for extracting the accelerometer data. Each course lasted for 3–4 min, approximately. Both the sensors that were used in the study sampled data at the frequency of 32 Hz. The complete data of all the 20 subjects had 237,760 samples for both the “On” and “Off” courses, respectively. Moreover, the data that was received from the accelerometer sensors contained a lot of noise. Therefore, for removing the noise, a fourth-order low pass Butterworth filter was used with a cutoff frequency of 15 Hz. The order of the filter was chosen based on the requirement of blocking the maximum noise, and the cutoff frequency was decided based on exploratory data analysis.

### 3.3. Feature Engineering

In the feature engineering step, two different sets of features were developed, namely statistical features and gait parameter features. For the calculation of features, data of each course of each patient was initially divided. Furthermore, data from each course were again grouped at the rate of 320 (10 s) samples. The computation process was implemented in such a way that the features were calculated for all the samples of a course for a particular data fold of 320 samples to maintain optimum variance between the feature values.

Table 3 shows all the statistical features that have been used in the study for the detection of the “On” and “Off” states in a patient suffering from PD. The implementation of statistical features in the proposed study provides us with the ability to identify some discriminant features, even if they lack obvious interpretability, but still seem to be important for determining intrinsic patterns for decision making. Table 4 explains some gait parameter features that have been derived from the course data based on the 320-sample data fold.

### 3.4. Machine Learning Algorithm and Evaluation Metrics

The development of the machine learning algorithms for the detection of “On” and “Off” states is very important to understand the patterns in the gait data of patients. Moreover, the algorithms leveraged in the work will also allow us to extract the semantic information from the data collected from the tri-axial accelerometer devices. In this work, multiple machine learning algorithms (Scikit-Learn) [32] were developed, and a comparative analysis was performed to determine the best possible algorithm based on the evaluation metrics. The initial hypothesis for the detection of the “On” and “Off” states from the gait data stated that the recall of both the classes must be higher than 85% and the misprediction rate in both the classes must be statistically equivalent. Table 5 shows all the hyperparameters that were used for the development of the machine learning model. 

The hyperparameters that were used for the machine learning model are critical to optimize the cost function and to increase the performance of the model. Therefore, initially, to choose the right set of hyperparameters, multiple iterations were performed by selecting specific hyperparameters using a trial and error technique. However, in the later segment, for the development of the final model for our web application’s production environment, we used a Bayesian Sequential Model-Based Optimization (SMBO) [33] method to choose the hyperparameters. Bayesian SMBO is an algorithm used for hyperparameter optimization that works on minimizing an objective function by creating a surrogate model (probability function) based on the evaluation results of the previous objective function. The basic objective function of the Bayesian SMBO is given by:

$$P\left(score|hyperparameters\right)=\frac{P(hyperparameters\;|score)P\left(score\right)}{P\left(hyperparameters\right)}$$

The surrogate model that is developed by the Bayesian SMBO is considered to be less expensive when than the main optimizer function [34]. Furthermore, the next set of evaluation results are selected by using the expected improvement criterion [35]. The criterion is defined as:

$$EI\left(x\right)=E\left(max\left(f\left(x\right)-f^{*},\;0\right)\right),$$

where *x* is the set of hyperparameter values and considered to have caused an improvement in the objective score of *f*(*x*) and f* is the maximum value of the objective score found in the process.

The basis of the development of all the machine learning algorithms is different, and therefore the feature selection for each machine learning algorithm is an important aspect that needs to be addressed. In the study, a Recursive Feature Elimination was performed on each machine learning algorithm to fetch the best set of features for discriminating between the “Off” class and “On” class. Table 6 shows the features that were used for the development of each machine learning algorithm by keeping the hyperparameters constant, as described in Table 5. 

The evaluation of the machine learning algorithms was carried out by considering accuracy, average recall, average precision, and average f1 score as the evaluation metrics. Accuracy in this particular task is used to understand the overall accuracy in both the “On” state and the “Off” state. In the current scope of the work, the recall is calculated for each class, where it provides information regarding the number of data samples that the model has correctly identified for a particular class. In this study, the average recall has been calculated to determine the average percentage of correct predictions (True Positive and True Negative) in each class. Moreover, the precision of the predictions has been calculated which determines the confidence of a particular prediction to belong to a particular class. Furthermore, for evaluation purposes, average precision was computed, which allowed us to identify the average of all the precision scores for each class. Finally, f1-score was computed, which provides a weighted average between both precision and recall of each class, and therefore penalizes the score for all the wrongly predicted samples of a particular class. Furthermore, during the learning procedure, the complete data of 20 subjects, i.e., 40 courses were divided into the ratio of 70:30 where data from 14 subjects were used for the training and data of six subjects for the validation/testing. As the developed model was used for the production environment, another concern that needs to be addressed is the generalizability of the model for incoming unseen data. Therefore, in this study, a “Leave One Subject Out (LOSO)”-based cross-validation was performed for all the 20 subjects for the best performing classifier to understand the generalizability of the model.

### 3.5. Web Application for the “On”/“Off” State Detection System

**Figure 4:**
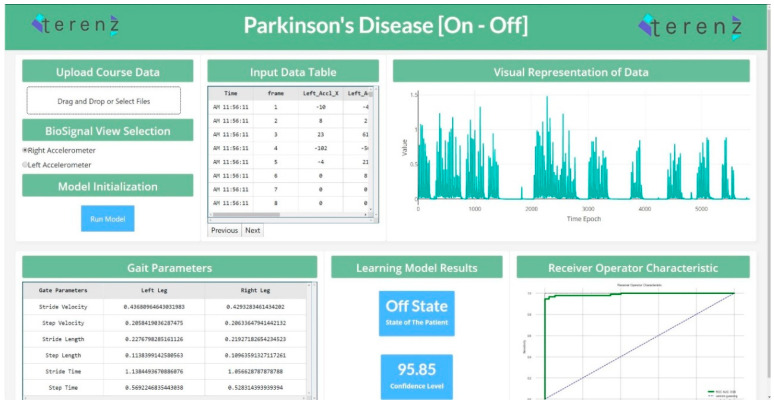
State Detection Web Application (https://parkinson-on-off.herokuapp.com/).

To productionize the machine learning models developed in the study, a web application was designed to detect the “On” and “Off” states. This application was developed using the Dash Framework (Plotly’s Python-based micro Web Framework using Flask as a Server). The application’s pipeline was designed to accommodate the raw data generated from the wearable device. This data is passed through the pre-processing step, and the system’s feature engineering routine. The preprocessing and feature engineering routine instantly clean, process and extract important features. In continuation, the extracted features were passed to the machine learning module, which performed the analysis and presented the predictions for each data segment. Figure 4 shows the web application for the detection of “On” and “Off” states. 

## 4. Results

**Figure 5:**
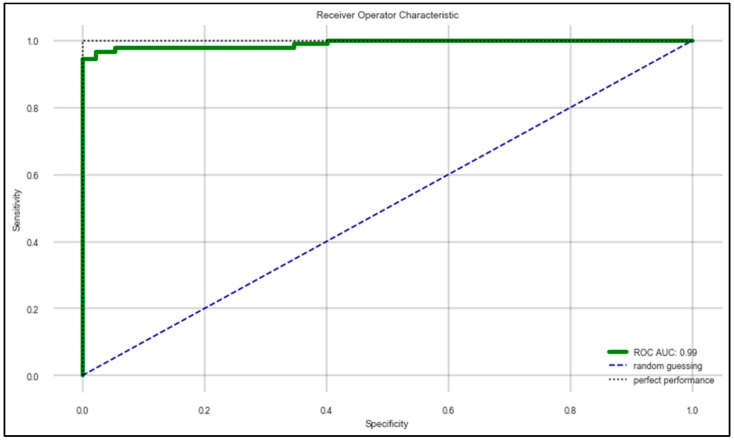
ROC–AUC of random forest classifier mentioned in Table 7.

The machine learning algorithms developed in the work provided some effective results in terms of determining the correct state of a patient suffering from PD. Table 7 provides a comparative analysis between all four machine learning algorithms that were used for the learning process and the evaluation metrics based on the randomized 70:30 split of the whole dataset using the hyperparameters mentioned in Table 5 and the features mentioned in Table 6. It can be observed that the random forest classifier performed the best in terms of classifying the “On” and “Off” states of PD patients. Figure 5 shows the ROC–AUC curve of the random forest classifier. For understanding the generalizability of the random forest classifier, a “Leave One Subject Out (LOSO)”-based cross-validation was performed for all the 20 subjects by keeping the same hyperparameters and features. It was observed that the model demonstrated an optimum generalizability in terms of predicting the data according to multiple test sets. The results are listed in Table 8.

**Figure 6:**
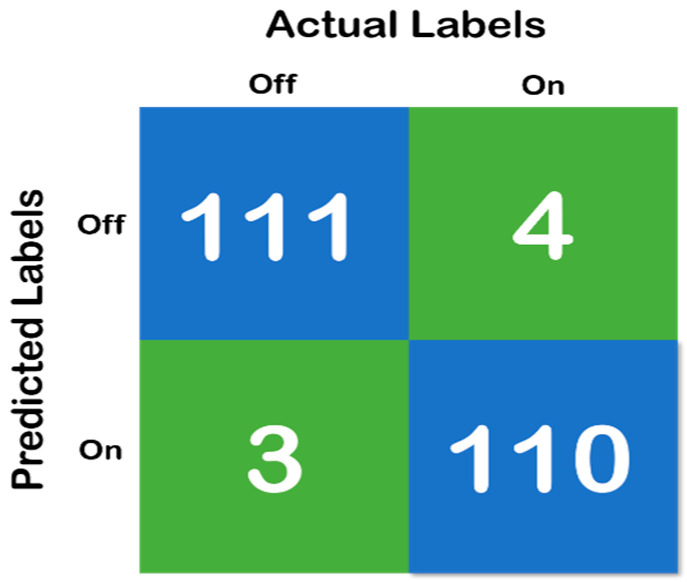
Confusion matrix of random forest classifier with 70:30 split, where the blue color represent the True Prediction and Green represents the Mispredictions.

**Figure 7:**
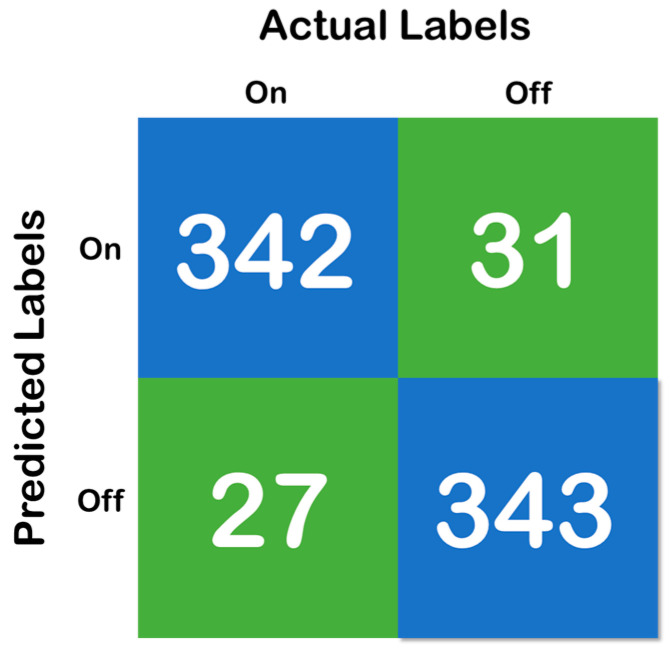
Confusion matrix of the cross-validation, where the blue color represent the True Prediction and Green represents the Mispredictions.

Figure 6, above, shows the confusion matrix that has been derived using the classification results of the test of the random forest data that has been developed with a training and testing split of 70:30. Moreover, Figure 7 shows the cross-validation confusion matrix of the random forest classifier which was obtained using the LOSO method. The confusion matrix that has been derived from the classification results on the test data and shows full accordance with the initial hypothesis that the number of correct predictions in each class needs to be above 85%—that iss the recall of each class needs to be above 85% and maintain a fixed classification rate across each class.

## 5. Discussion

This study developed a framework for detecting the “On” and “Off” states for monitoring PD patients by leveraging the wearable accelerometer data, instead of the conventional method of collecting the data from a patient diary either written by the caregiver or by the patient, which is used by the physicians for regulating medication therapy [35]. A variety of features that were obtained from the accelerometer signals were fed as inputs to differentiate between two different states with a low rate of misclassification. In the past, researchers have used accelerometer signal-based models for quantifying the gait parameters, and those data were used for classifying different motor symptoms such as freezing of gait and shuffling of gait [14,22]. However, the wearable accelerometer signal-based model, including spatiotemporal gait features, has not been studied in detail for detecting medication effects. The spatiotemporal gait features that were quantified based on our earlier study [22], and which are considered in this study, were treated as the most important parameters based on the results of our past study [22], as well as past studies performed by other researchers [36,37,38,39,40]. Four machine learning techniques have been used for classifying the “On” state and the “Off” state. Among them, random forest performed the best, and provided an accuracy of 96.72%. The developed model was validated by the report provided by the neurologist recorded in real time. Our model performance was good, with an average recall of 97.35% and average precision of 96.92%, although we trained our model with a small number of data. This above observation laid out a strong recommendation that the developed model could be a candidate to be used in home environments for detecting the “On” and “Off” states.

In this research, we have investigated the feasibility of using wearable accelerometers in both the knees for the classification of “On” and “Off” states. Although past studies cited that the placement of the sensor has a huge role to play in the performance of the results, in this study, we placed the sensor at the knee because our past research on detecting “FoG” [22] was successful when using knee-based signals.

There have been studies that have tried to monitor motor complications in PD [1,2,3]. Despite these innovatory studies, a wearable sensor for motor complications has not yet been applied in the clinical field due to a lack of acceptable clinical validation. Most studies use a frequency-based approach from the accelerometer, and therefore there may be technical bias in reflecting real-world environments. Parkinsonian symptoms vary between individuals and show inconsistent clinical presentations. The machine learning techniques have been emerging in recent years, and they provide the possibility of overcoming this limitation. Moreover, we postulated that the specification of Parkinsonian symptoms such as tremors, gait disorder, and bradykinesia is necessary to increase the accuracy. Our study is the first step in developing an algorithm for discriminating gait symptoms in “On”/“Off” states using a machine learning technique based on the data collected through a wearable sensor. We assume that our algorithm could be a potential candidate as a clinical monitoring tool to assess free-living environments.

The novelties of this study are as follows. (1) This is the first study that used statistical features and spatiotemporal gait features for the classification of “On” and “Off” states. (2) This is the first study that used knee-based wearable acceleration signals for detecting “On” and “Off” states. (3) This is the first study in which a robust machine learning model has been developed using the raw accelerometer signals for classifying “On” and “Off” states. (4) This study also contained a web application in which the complete process could be experienced, starting from the input of the data to the data distribution, and resulting in a single web page within a few seconds.

Our study has been compared with various state-of-the-art models that used wearable accelerometer signals for differentiating “FoG” from “no FoG”, shuffling of gait from no shuffling of gait, PD group from healthy group, mediation state (“On” and “Off”), and PD and other neurological diseases. The developed system in this study was found to outperform all the previous studies that use PD-related classification. A comparative performance analysis of these studies is shown in Table 9.

Moreover, for analyzing the performance of the model for productionizing it in a clinical setting, the review of Type I (false negatives: the person is in the “On” stage but the model predicted it as “Off”) and Type II (false positives: the person is in the “Off” stage but the model predicted it as “On”) errors are of paramount importance. Normally, in trivial machine learning projects, a certain type of error can be rejected during the hypothesis testing. However, in the present study, both the errors are of paramount importance because of the following cases:

1. If the patient is in the “On” state but the model predicts it as the “Off” state (Type I error), then the doctor might conclude that the patient is in the “Off” state and his gait paramters are fine, and it might result in wrongly reducing the medicinal dosage.
2. If the patient is in the “Off” state but the model predicts it as the “On” state (Type II error), then the doctor might conclude that the patients is in the “On” state and his gait parameters are still very low, and it might result in wrongly increasing the medicinal dosage.

Therefore, both the errors (Type I and II) must be considered when analyzing the models. Moreover, concerning the model developed in the present study and the results depicted in Table 8 and Figure 6, respectively, it can be found that the error rates are low. We can observe in each sample depicted in Table 8 and Figure 6, accounting for 10 s of data, i.e., 320 samples, that the mispredictions for the “On” data cycle occur in the very beginning of the acquired data samples. For the “Off” data cycle, it occurs at the middle of or in between the processes. Therefore, for the “On” state, it can be considered that the first 10–15 s is typically a heads up time where the subject gears up for the process. For the “Off” state, mispredictions are typically in the samples acquired halfway into the process, which means that the subject becomes stable (High Gait Values) after conducting the exercise for some time, but gets tired at the end. However, the specificity, sensitivity (recall), precision (positive predicted value) and diagnostic odds ratio (DOR) are considered to be the major metrics to determine the reliability of a clinical software system. Therefore, based on the confusion matrix depicted in Figure 6, it was found that the specificity of the system is 0.96, the sensitivity is 0.982, PPV is 0.972 and the DOR is 1027. Looking at the aforementioned results, it can be duly observed that the system developed in the work is very much reliable for its implementation in clinical settings. However, as a limitation of the work, it also must be noted that only the gait parameters are not sufficient for the detection of “On” and “Off” states in Parkinson’s disease.

## 6. Conclusions

In this study, the detection of “On” and “Off” states based on gait signals and using a wearable accelerometer were proposed, and machine learning-based techniques have been used to automate the classification of two states using the wearable accelerometer signals. The methodology proposed in this research signifies the efficacies of using wearable accelerometer-based data in combination with machine learning techniques for the auto-detection of different medication states in PD. The algorithm that was developed in this study used the data of 20 subjects suffering from PD. The developed algorithm was able to detect the “On” and “Off” states, and was also validated by the report of the neurologist. This research contained a unique combination of statistical features and gait features that are fed as inputs to the machine learning classifiers. Among the four machine learning techniques used in this study, random forest outweighs all the other classifiers, detecting the two states with an accuracy of 96.72%, recall of 97.35%, and precision of 96.92%. Some of the new findings observed in this study are as follows: (1) only accelerometer signals were able to detect the medication state in combination with machine learning techniques. (2) The knee-based placement of wearable devices mentioned in this research was able to perform better compared to the combination of wrist- and ankle-based wearable sensors [42]. This study has the following limitations: (1) all data were obtained using a wearable accelerometer while the subject was performing tasks only in the laboratory environment. (2) This study is mainly intended for detecting the “On” and “Off” states, and is not intended to assess other motor symptoms such as “FoG” and shuffling of gait.

Possible directions for future work include the development of a more robust model with more data. The performance of the model in the home environment is yet to be verified. From the current observations mentioned in this study, it is recommended that the developed algorithm could perform well in home environments in real-life situations.

## Figures and Tables

**Table 1:** Related works.

Reference	Input Signals	Features	Classifiers	Outcomes	Performance Indices
[20]	Accelerometer, gyroscope	Temporal, Spectral features	DT,SVM,DA, RF,k-NN	Accuracy 85.5%	Accuracy, Precision, Recall
[21]	Accelerometer	Spatiotemporal features	Support Vector Regression	Accurcay 90%	Accuracy
[22]	Accelerometer	Spatiotemporal features	SVM,NB,k-NN,DT	Accuracy 88%	Accuracy
[23]	Accelerometer, Gyroscope, Magnetometer	One Dimensional CNN features	CNN	Accuracy 93.4%	Accuracy
[24]	Accelerometer	Statistical Features	SVM,k-NN,NLP	Accuracy 97.4%	Accuracy
[25]	Accelerometer, Electromyography	Time Domain Features	DT,k-NN,LDA,SVM, Boosted tree Classifier, Bagged Tree Classifier	Accuracy 99.6%	Accuracy
[26]	Accelerometer, Gyroscope, Magnetometer	Spatiotemporal features	Logistic Regression and RF	Sensitivity 86% Specificity 90%	Sensitivity, Specificity
[27]	Accelerometer	Frequency Domain Features	LSTM,SVM	Accuracy 83.38%	Accuracy
[28]	Speech Signals	Voice features	SVM,ANN,CART	Accuracy 93.84%	Accuracy

**Table 2:** Demographics of Parkinson’s disease (PD) Patients.

Parameter	Value
Age	60.25 ± 9.54
M/F	12/8
Disease duration (month)	144.91 ± 166.71
BMI	22.87 ± 3.04
Levodopa Equivalent Doses(LEDD)	985.69 ± 457.49
H&Y stage “On” State	1.70 ± 0.66
H&Y stage “Off” State	2.65 ± 0.71
UPDRS part III “On” State	15.80 ± 10.13
UPDRS part III “Off” State	36.20 ± 15.06

**Table 3:** Statistical features.

Feature	Equation	Description
Mean	$\bar{X}=\frac{1}{n}\overset{n}{\underset{i=0}{\sum }}\left(X_{i}\right)$	The mean of the feature was calculated for each subject for all the samples in each fold, i.e., 320 samples.
Standard Deviation	$\sigma =\sqrt{\frac{1}{N}\overset{N}{\underset{i=1}{\sum }}{\left(X_{i}-\bar{X}\right)}^{2}}$	The standard deviation of the signal is calculated for each subject for all the samples in each fold, i.e., 320 samples.
Median Absolute Deviation	$MAD=median\left(\left|X_{i}-\tilde{X}\right|\right)$ where, $\tilde{X}$ is the median of X	The median absolute deviation was calculated each subject for all the samples in each fold, i.e., 320 samples.
Minimum	min(X)	The minimum of the signal was for calculated each subject for all the samples in each fold, i.e., 320 samples.
Maximum	max(X)	The maximum of the signal was for calculated each subject for all the samples in each fold, i.e., 320 samples.
Energy Measure	$E_{x}=\frac{1}{n}\overset{n}{\underset{i=0}{\sum }}{\left|x\left[i\right]\right|}^{2}$	The energy measure of the signal was calculated for each subject based on all the samples in each fold, i.e., 320 samples.
Inter Quartile Range	$IQR=median\left(Q_{3}\right)-median\left(Q_{1}\right)$	The interquartile range was calculated on each subject by considering each fold that is 320 samples of data as the complete set.
Signal Magnitude Area	$SMA=\frac{1}{n}\overset{n}{\underset{i=0}{\sum }}(\left|X_{i}\right|+\left|Y_{i}\right|+|Z_{i}|)$	The signal magnitude area is been calculated by considering all the axis of the accelerometer and gyroscope and is calculated for each subject by considering the samples in each fold.
Skewness	$Skew\left(X\right)=\frac{n}{\left(n-1\right)\left(n-2\right)}\overset{n}{\underset{i=0}{\sum }}\left(\frac{X_{i}-\bar{X}}{s}\right)$	The skewness for all the samples of a fold for a particular subject was calculated based on the Fisher-Pearson standardized moment coefficient
Kurtosis	$Kurt\left(X\right)=\frac{E\left[{\left(X-\bar{X}\right)}^{4}\right]}{std{\left(X\right)}^{2}}-3$	The kurtosis (peakedness) is calculated for individual signals.

**Table 4:** Gait Parameter Based Feature.

Feature	Description
Step Length	The distance between the heel of one foot to the heel of the other foot.
Stride Length	The distance covered between two steps, one with each foot
Step Time	The time taken by a person for completing one step
Stride Time	The time taken by a person to complete one stride length
Step Velocity	The speed at which a step length is covered.
Stride Velocity	The speed at which a stride length is covered.

**Table 5:** State detection: classifiers and specifications.

Classifier	Specifications
Random Forest	n_estimators: 500, criterion = ‘gini’*,* max_depth:8, min_samples_split = 8, min_samples_leaf = 10
Support Vector Machine	kernel = ‘rbf’, degree = 3, gamma = ‘auto_deprecated’, C = 1.0, tol = 0.001, cache_size = 200
K–Nearest Neighbors	n_neighbors = 50, weights = ‘uniform’, algorithm = ‘auto’, leaf_size = 40, p = 2, metric = ‘minkowski’
Naïve Bayes	Gaussian

**Table 6:** Feature selection for all the classifiers.

Features	RFC	SVM	KNN	NB
Mean (Left Knee)	Used	Removed	Removed	Used
Standard Deviation (Left Knee)	Used	Used	Removed	Used
Median Absolute Deviation (Left Knee)	Removed	Used	Used	Used
Minimum (Left Knee)	Used	Used	Used	Used
Maximum (Left Knee)	Used	Removed	Used	Removed
Energy Measure (Left Knee)	Used	Used	Used	Used
Inter Quartile Range (Left Knee)	Used	Used	Used	Removed
Signal Magnitude Area (Left Knee)	Used	Used	Removed	Removed
Skewness (Left Knee)	Removed	Removed	Used	Used
Kurtosis (Left Knee)	Used	Used	Used	Removed
Step Length (Left Knee)	Used	Used	Removed	Used
Stride Length (Left Knee)	Removed	Removed	Used	Used
Step Time (Left Knee)	Used	Removed	Removed	Used
Stride Time (Left Knee)	Removed	Used	Used	Used
Step Velocity (Left Knee)	Used	Used	Removed	Used
Stride Velocity (Left Knee)	Used	Removed	Used	Used
Mean (Right Knee)	Used	Used	Removed	Removed
Standard Deviation (Right Knee)	Used	Used	Used	Used
Median Absolute Deviation (Right Knee)	Used	Used	Used	Used
Minimum (Right Knee)	Removed	Removed	Used	Used
Maximum (Right Knee)	Used	Used	Used	Removed
Energy Measure (Right Knee)	Used	Removed	Used	Used
Inter Quartile Range (Right Knee)	Removed	Used	Removed	Removed
Signal Magnitude Area (Right Knee)	Used	Removed	Used	Used
Skewness (Right Knee)	Used	Used	Used	Used
Kurtosis (Right Knee)	Removed	Used	Used	Used
Step Length (Right Knee)	Used	Used	Removed	Removed
Stride Length (Right Knee)	Removed	Used	Used	Used
Step Time (Right Knee)	Used	Removed	Removed	Removed
Stride Time (Right Knee)	Removed	Used	Used	Removed
Step Velocity (Right Knee)	Used	Removed	Removed	Used
Stride Velocity (Right Knee)	Removed	Used	Used	Removed

**Table 7:** Comparative analysis of multiple classifiers.

Classifier	Accuracy	Average Recall	Average Precision	Average F1 Score	ROC–AUC Score	95% CI Lower Limit	95% CI Upper Limit	Average Training Time
RF	0.96	0.97	0.96	0.97	0.99	0.95	1.0	5.46 s
SVM	0.93	0.92	0.93	0.93	0.94	0.92	0.98	7.14 s
KNN	0.86	0.84	0.85	0.85	0.87	0.81	0.89	0.21 s
Naïve Bayes	0.88	0.86	0.85	0.86	0.87	0.82	0.91	0.15 s

**Table 8:** Confusion catrix values for each subject as tests of best performing classifier (RF).

Test Set	True Positive “On”-“On”	False Positive “On”-“Off”	False Negative “Off”-“On”	True Negative “Off”-“Off”	Total Samples “On”/”Off”	Aggregated Samples (10 s) “On”/“Off”
Subject 1	15	2	1	15	5370/5217	17/16
Subject 2	17	1	1	16	5774/5471	18/17
Subject 3	14	2	1	17	5138/5883	16/18
Subject 4	17	3	2	17	6264/6214	20/19
Subject 5	15	2	2	19	5508/6749	17/21
Subject 6	16	1	1	21	5438/6883	17/22
Subject 7	19	1	2	17	6282/5972	20/19
Subject 8	17	4	1	16	6640/5347	21/17
Subject 9	17	1	2	17	5845/5987	18/19
Subject 10	19	1	2	15	6321/5465	20/17
Subject 11	18	0	1	19	5792/6321	18/20
Subject 12	17	1	2	19	5795/6784	18/21
Subject 13	19	2	1	15	6795/5039	21/16
Subject 14	13	1	2	19	4499/6742	14/21
Subject 15	19	1	0	17	6461/5398	20/17
Subject 16	21	2	0	17	7293/5418	23/17
Subject 17	14	2	2	18	5007/6472	16/20
Subject 18	20	1	1	18	6608/6128	21/19
Subject 19	16	1	2	16	5510/5890	17/18
Subject 20	20	2	1	15	6912/5128	22/16
Total	342	31	27	343	119,252/118,508	373/370

**Table 9:** A comparison of our results with state-of-the-art models’ work for activity detection.

Author	Objective	Location of Sensors	Accuracy (%)
Our Work	“On”/“Off” detection	Knee	96.72
Aich et al. [22]	“FoG”/“No FoG” detection	Knee	88.00
Aich et al. [41]	PD/HOG classification	Knee	88.46
Hssayeni et al. [42]	“On”/“Off” detection	Wrist and Ankle	90.50
Rehman et al. [18]	PD/HC classification	Lower back	87.83
